# Analysis of the relationship between phenotypes and genotypes in 60 Chinese patients with propionic acidemia: a fourteen-year experience at a tertiary hospital

**DOI:** 10.1186/s13023-022-02271-3

**Published:** 2022-03-24

**Authors:** Yi Liu, Zhehui Chen, Hui Dong, Yuan Ding, Ruxuan He, Lulu Kang, Dongxiao Li, Ming Shen, Ying Jin, Yao Zhang, Jinqing Song, Yaping Tian, Yongtong Cao, Desheng Liang, Yanling Yang

**Affiliations:** 1 Department of Pediatrics, Peking University First Hospital, Beijing, 100034 China; 2 Department of Clinical Laboratory, China-Japan Friendship Hospital, Beijing, 100029 China; 3 Department of Endocrinology and Genetic, Beijing Children’s Hospital, Capital Medical University, Beijing, 100045 China; 4 Department of Pediatrics, The First Affiliated Hospital of Zhengzhou University, Zhengzhou, 450052 China; 5 Department of Endocrinology and Genetic, Henan Children’s Hospital, Zhengzhou, 450053 China; 6 Translational Medicine Center, Chinese PLA General Hospital, Beijing, 100853 China; 7 School of Life Sciences, Central South University, Changsha, 410000 China

**Keywords:** Propionic acidemia, *PCCA* gene, *PCCB* gene, Propionyl-CoA carboxylase

## Abstract

**Background:**

Propionic acidemia is a severe inherited metabolic disorder, caused by the deficiency of propionyl-CoA carboxylase which encoded by the *PCCA* and *PCCB* genes. The aim of the study was to investigate the clinical features and outcomes, molecular epidemiology and phenotype-genotype relationship in Chinese population.

**Methods:**

We conducted a retrospective study of 60 Chinese patients diagnosed at Peking University First Hospital from 2007 to 2020. Their clinical and laboratory data were reviewed. The next-generation sequencing was conducted on blood samples from 58 patients.

**Results:**

Only 5 (8.3%) patients were identified by newborn screening. In the rest 55 patients, 25 had early-onset (≤ 3 months) disease and 30 had late-onset (> 3 months) disease. Neurological abnormalities were the most frequent complications. Five cases detected by newborn screening had basically normal development. Nine (15%) cases died in our cohort. 24 patients (41.4%) harbored *PCCA* variants, and 34 (58.6%) harbored *PCCB* variants. 30 (11 reported and 19 novel) variants in *PCCA* and 28 (18 reported and 10 novel) variants in *PCCB* mere identified. c.2002G>A and c.937C>T in *PCCA*, and c.838dupC in *PCCB* were the most common variants in this cohort, with the frequency of 13.9% (6/44 alleles), 13.9% (6/44 alleles) and 12.5% (8/64 alleles), respectively. There was no difference in clinical features and outcomes between patients with *PCCA* and *PCCB* variants. Certain variants with high frequencies and homozygotes may be associated with early-onset or late-onset propionic acidemia.

**Conclusions:**

Although the genotype–phenotype correlation is still unclear, certain variants seemed to be related to early-onset or late-onset propionic acidemia. Our study further delineated the complex clinical manifestations of propionic acidemia and expanded the spectrum of gene variants associated with propionic acidemia.

**Supplementary Information:**

The online version contains supplementary material available at 10.1186/s13023-022-02271-3.

## Background

Propionic acidemia (OMIM #606054) is a serious inherited metabolic disorder caused by the deficiency of mitochondrial biotin-dependent enzyme propionyl-CoA carboxylase (EC 6.4.1.3). The worldwide incidence of propionic acidemia is approximately 1 in 100, 000 to 1 in 50, 000 live births, but varied in region and ethnicity [1, 2]. In China, although no national data on the disease prevalence were reported, the results of newborn screening in Zhejiang province revealed a low prevalence of 0.32 in 100, 000 newborns [3].

Propionyl CoA carboxylase consists of six α- and six β-subunits, encoded by *PCCA* (OMIM #232000) and *PCCB* (OMIM #232050) genes. Pathogenic biallelic variants of either *PCCA* or *PCCB* gene lead to deficient enzyme activity and toxic metabolites accumulation, resulting in damages of multiple organs and systems. More than 100 *PCCA* and *PCCB* variants have been reported, while the hotspot variants and variant spectrum are widely different among regions [4]. Up to now, no clear relationship was confirmed between clinical phenotypes and genotypes in propionic acidemia [4–6].

Due to different degrees of enzyme deficiency, the clinical features of propionic acidemia are heterogenous, ranging between asymptomatic forms and severe forms, like death in the neonatal period or later due to the metabolic crisis or cardiac complications [7]. Early detection by newborn screening is an effective approach to identify patients at early stage [8]. Previous researches suggested that newborn screening can reduce the short-term mortality of propionic acidemia [9], while the health gain of newborn screening for overall outcome of propionic acidemia, including the number of metabolic crises and long-term complications, may be limited [10, 11].

In the present study, we investigated the process of diagnosis, treatment and prognosis of 60 Chinese patients with propionic acidemia to analyze their clinical, biochemical and genetic features. The aim of the study was to further delineate the spectrums of clinical manifestations and gene variants in patients with propionic acidemia, and explore the phenotype-genotype correlation.

## Materials and methods

### Patients

This study was approved by the Hospital Institution Committee in accordance with the Declaration of Helsinki. Written informed consents were obtained from all the patients’ legal guardians. Sixty patients (36 males and 24 females) were admitted to Peking University First Hospital between January 2007 and December 2020. They came from fifty-six non-consanguineous families from sixteen provinces of China. Their clinical and laboratory data at onset and follow-up were collected.

The diagnosis of propionic acidemia was established by elevated blood propionylcarnitine, urine methylcitrate and 3-hydroxypropionic acid levels, and normal urine methylmalonic acid concentration. In this study, propionic acidemia was divided into early-onset form (age of onset ≤ 3 months) and late-onset form (age of onset > 3 months) [12].

### Metabolic examinations

Newborn screening and high-risk screening were performed by the analysis of blood amino acid and acylcarnitine profiles (NeoBase Non-derivatized MSMS Kit, PerkinElmer, Massachusetts, USA), using liquid chromotography-tandem mass spectrometry (LC-MS/MS, API3200 or Triple Quad 4500, Applied Biosystems, CA, USA) [13, 14]. ChemoView software was used for the calculation of metabolite concentrations. The reference range for propionylcarnitine was 1.00–4.00 μmol/L.

For the differential diagnosis of organic acidemias, urine organic acids were determined using gas chromatography-mass spectrometry (GC–MS, GCMS-QP2010 plus, Shimadzu, Kyoto, Japan) as previously described [15, 16]. The Inborn Errors of Metabolism Screening System software was used for data processing and analysis. The normal mean values of urine methylcitrate and 3-hydroxypropionic acid were both 0.2 mmol/mmol creatinine, both with a range from 0 to 1.1 mmol/mmol creatinine.

### Treatment

The treatment included acute management and long-term management [11, 17]. During the acute phase of metabolic decompensation, protein restriction, high-calorie intake, intravenous infusion of l-carnitine (100–300 mg/kg/d), glucose and electrolytes to correct metabolic acidosis, and symptomatic treatments were given to the patients. Many patients had low blood arginine levels (below 10 µmol/L), and arginine (100–200 mg/kg/d) instead of carbaglu (not available in China) was used to lower blood ammonia.

Long-term management included oral l-carnitine (30–100 mg/kg/d), dietary management including restriction of natural proteins, supplementation of medical foods or special formulas (without isoleucine, valine, methionine and threonine) and high-carbohydrate and high-fat diets, and treatments for complications. For dietary management, the total protein intake was kept in 2.5–3.0 g/kg/d for infants, 30–40 g/d for children and 50–65 g/d for adults; natural protein intake was controlled within 1.2–1.8 g/kg/d in the infants under 6 months of age, 0.6–1.2 g/kg/d in the patients aged 6 months to 7 years, 0.5–1.0 g/kg/d in the patients aged 7 years to 18 years, and 0.4–0.8 g/kg/d in the patient above 18 years old; many patients were prescribed the medical foods at a dosage of 0.6–1.2 g/kg/d for infants, 0.3–0.6 g/kg/d for children, and 0.2–0.4 g/kg/d for adults, and the intake was also adjusted for individual tolerance, growth spurts and minor illnesses. Besides, liver transplantation was an option for patients with frequent metabolic decompensation.

### Genetic testing

Next-generation sequencing (NGS) was performed for variant screening. Briefly, genomic DNA was extracted from peripheral blood samples or dried blood spots of patients, then prepared for high-throughput sequencing. The DNA was sequenced on Illumina Hiseq2500 (Illumina, San Diego, USA) using a designed panel of 142 inherited metabolic disorder associated genes, including *PCCA* and *PCCB*. After discarding low quality reads from raw data, clean sequencing reads were aligned to the reference human genome (hg19). Variants were compared with Human Gene Mutation Database (HGMD), in-house database, 1000 Genomes, ExAC database (http://exac.broadinstitute.org/) and gnomAD database (http://gnomad.broadinstitute.org/). The pathogenicity of novel variants was assessed according to the American College of Medical Genetics and Genomics and the Association for Molecular Pathology (ACMG/AMP) standards and guidelines [18].

After suspected pathogenic variants were detected by NGS, Sanger sequencing, multiplex ligation-dependent probe amplification or quantitative real-time PCR was conducted on DNA samples of patients and parents for further confirmation and familial co-segregation analyses.

### Statistical analysis

Continuous variables were expressed as mean ± standard deviation (SD) or median (range). Categorical variables were presented as frequencies and percentages. Student *t* tests and Mann–Whitney U tests were used to compare continuous variables between groups. The categorical variables between groups were compared using Chi-squared tests. SPSS 20.0 (IBM, Armonk, USA) was used for statistical analysis, and *P* < 0.05 was considered statistically significant.

## Results

### Clinical features and laboratory examinations

Among the 60 patients with propionic acidemia, 5 (8.3%) were found by newborn screening in the 7th day to 11th day of life. The rest 55 (91.7%) cases were clinically diagnosed at the age of 5 days to 4 years. The onset age of the 55 cases was from 2 h after birth to 3.5 years, with a median age of 5 months. Among them, 25 cases had early onset and 30 cases had late onset. 23 (38.3%) cases had symptoms during the neonatal period.

The initial clinical manifestations of 60 patients were significantly different. Five children detected by newborn screening were almost asymptomatic. While the 55 patients who were clinically diagnosed had varied symptoms, including lethargy (42 cases, 70.0%), hypotonia (38 cases, 63.3%), vomiting (25 cases, 41.7%), poor feeding (23 cases, 38.3%), development delay (19 cases, 31.7%), seizures (18 cases, 30.0%) and dyspnea (16 cases, 26.7%). The patients developed different degrees of multi-organ complications, which were summarized in Table 1. The main complications were intellectual disability (36 cases, 60.0%), hematological abnormalities including anemia, pancytopenia and thrombocytopenia (31 cases, 51.7%), epilepsy (25 cases, 41.7%), skin lesions (19 cases, 31.7%), which were suspected of essential amino acid deficiency (many patients may have been over-restricted in protein intake by their local hospitals), intermittent vomiting (18 cases, 30.0%) and liver involvement including hepatomegaly and liver dysfunction (15 cases, 25.0%). Four patients developed cardiac diseases. One case with early-onset propionic acidemia had acute heart failure. One case with late-onset propionic acidemia had cardiac hypertrophy. The rest two cases were brothers, both of whom had late-onset propionic acidemia and developed long QT syndrome. However, there was no significant difference in the frequencies of complications between patients with early- and late-onset propionic acidemia in the follow-up period of this study (Table 1).

**Table 1:** Complications of 60 Chinese patients with propionic acidemia

Complications	Total^a^ (60)		Early-onset (25)		Late-onset (30)		*P* value^f^
	n	%	n	%		%	
Skin lesions	19	31.7	7	28.0	12	40.0	0.351
Neurological involvement							
Intellectual disability	36	60.0	16	64.0	20	66.7	0.836
Seizures	25	41.7	8	32.0	17	56.7	0.114
Hearing impairment^b^	3	5.0	1	4.0	2	6.7	1.000
Hematological abnormalities							
Anemia	23	38.3	12	48.0	10	33.3	0.269
Pancytopenia	9	15.0	5	20.0	4	13.3	0.765
Thrombocytopenia	2	3.3	2	8.0	0	0	0.202
Digestive system abnormalities							
Intermittent vomiting	18	30.0	5	20.0	12	40.0	0.110
Liver involvement^c^	14	25.0	7	28.0	7	23.3	0.692
Renal dysfunction^d^	2	3.3	2	8.0	0	0	0.202
Cardiac diseases^e^	4	6.7	1	4.0	3	10.0	1.000
Death	9	15.0	7	28.0	2	6.7	0.078
Age at the last follow-up (years, median and range)	3.0 (0.2–19.0)		1.4 (0.2–10.0)		3.3 (0.7–19.0)		0.021

^a^The five patients found by newborn screening were not classified as early-onset (onset age ≤ 3 months) or late-onset (onset age > 3 months) propionic acidemia due to no obvious symptoms

^b^Hearing impairment referred to bilateral delay of auditory brainstem response

^c^Liver involvement included hepatomegaly and liver dysfunction (increased liver enzymes)

^d^Renal dysfunction referred to acute renal failure

^e^Cardiac diseases included long QT syndrome and heart failure

^f^Chi-square test and Mann–Whitney U test were used for comparisons between early- and late-onset propionic acidemia

Common laboratory abnormalities included metabolic acidosis with increased anion gap (37 cases, 61.7%), hyperammonemia (26 cases, 43.3%), hemocytopenia (19 cases, 31.7%), and hyperglycinemia (15 cases, 25.0%). The maximum value of plasma ammonia was 798 μmol/L (reference range 0–60 μmol/L). Brain MRI examinations were performed in 34 patients. 23 cases had imaging abnormalities, including bilateral basal ganglia damages (14 cases), white matter changes (3 cases), cerebral atrophy (3 cases), intracranial hemorrhage (1 case), medullary lesions (1 case) and scattered lesions in cerebellar hemispheres (1 case).

### Follow-up and clinical outcome

Five patients detected by newborn screening were 4 months to 16 years old at the last follow-up, with normal development and no obvious symptoms. 9 (15%) patients died at the age of 10 days to 19 years. Seven early-onset patients died of acute metabolic decompensation. Two late-onset patients died at the age of 7.5 and 19 years due to acute metabolic decompensation and long QT syndrome, respectively. The remaining 46 cases were significantly relieved of clinical symptoms after individualized dietary management, drug therapy and supportive treatment, but they still had varying degrees of intellectual or motor development delay.

In our cohort, 3 cases received liver transplants from parent donors at the age of 2 years, 2.8 years and 5.8 years, respectively. Their blood propionylcarnitine declined dramatically from 32.0–130.0 μmol/L to 9.0–37.5 μmol/L after transplantation. At the last follow-up, they were 3.5 years to 7 years old, with oral L-carnitine treatment and normal diet.

### *PCCA* and *PCCB* variants

Fifty-eight (96.7%) patients and their parents underwent genetic analysis. All patients harbored homozygous or compound heterozygous variants in *PCCA* or *PCCB*, except for one patient with only heterozygous variant in *PCCB*. Among them, 24 (41.4%) patients had *PCCA* variants, including two pairs of siblings. 34 (58.6%) patients had *PCCB* variants, also including two pairs of siblings. The biallelic variants and clinical features of the 60 individuals were detailed in Additional file 1.

A total of 30 *PCCA* variants were detected, including 11 reported and 19 novel variants (Table 2). All the novel variants were pathogenic or likely to be pathogenic according to the ACMG/AMP standards, and uploaded to ClinVar (https://www.ncbi.nlm.nih.gov/clinvar, submission ID: SUB5596791). Among the 24 patients with *PCCA* variants, one case was homozygous for c.2002G>A variant, and the remaining 23 cases were compound heterozygous. In our cohort, c.2002G>A and c.937C>T were the most frequent *PCCA* variants, both with a frequency of 13.9% (6/44 alleles).

**Table 2:** Details of *PCCA* variants detected in the 60 Chinese patients

Novel/ reported	No	Exon	Nucleotide alteration^a^	Protein alteration	Allele frequency	ACMG/AMP grading		HGMD/Clinvar accession	Population frequency (GnomAD, v2.1.1)
						Classification	Evidence code		
Novel	1	Exon 1	c.2T>A	p.M1K	1/44	P	PVS1, PM2, PP1, PP3, PP4	N/A	N/A
	2	Exon 1	c.43G>T	p.G15*	1/44	P	PVS1, PM2, PP1, PP3, PP4	N/A	N/A
	3	Exon 2	c.130_131delinsTATT	p.C44Yfs*3	1/44	P	PVS1, PM2, PP1, PP3, PP4	N/A	N/A
	4	Exon 5	c.305delA	p.H102Lfs*2	2/44	P	PVS1, PM2, PP1, PP3, PP4	N/A	N/A
	5	Exon 5	c.331_332delTG	p.V112Wfs*7	1/44	P	PVS1, PM2, PP1, PP3, PP4	N/A	N/A
	6	Exon 7	c.524G>A	p.G175D	1/44	LP	PM2, PM5, PP1, PP3, PP4	N/A	N/A
	7	Exon 9	c.683G>T	p.G228V	1/44	LP	PM2, PM5, PP1, PP3, PP4	N/A	N/A
	8	Exon 9–22	exon 9–22 del	N/A	1/44	LP	PVS1, PM2, PP1, PP4	N/A	N/A
	9	Exon 10	c.734C>G	p.S245*	2/44	LP	PVS1, PM2, PP1, PP3, PP4	N/A	N/A
	10	Exon 10	c.803G>T	p.R268L	1/44	LP	PM2, PM5, PP1, PP3, PP4	N/A	N/A
	11	Exon 11	c.869G>A	p.C290Y	1/44	LP	PM2, PM5, PP1, PP3, PP4	N/A	N/A
	12	Exon 11	c.872C>T	p.S291L	1/44	LP	PM2, PM5, PP1, PP3, PP4	N/A	N/A
	13	Exon 12	c.917dupT	p.L308Ffs*35	1/44	P	PVS1, PM2, PP1, PP3, PP4	N/A	N/A
	14	Exon 13	c.1066G>C	p.V356L	1/44	LP	PM2, PM3, PP1, PP3, PP4	N/A	N/A
	15	Exon 13	c.1074_1076delTCC	p.359del	1/44	LP	PM2, PM4, PP1, PP3, PP4	N/A	N/A
	16	Exon 15	c.1328_1329insT	p.Y444Lfs*3	1/44	P	PVS1, PM2, PP1, PP3, PP4	N/A	N/A
	17	Exon 22	c.1919delC	p.R641Dfs*6	1/44	P	PVS1, PM2, PP1, PP3, PP4	N/A	N/A
	18	Exon 23	c.2077A>T	p.M693L	1/44	LP	PM2, PM3, PP1, PP3, PP4	N/A	N/A
	19	Exon 24	c.2162_2163insAAGG	p.D722Rfs*12	1/44	P	PVS1, PM2, PP1, PP3, PP4	N/A	N/A
Reported	20	Intron 1	c.105+1G>A	splicing	1/44	N/A	N/A	VCV000218251	N/A
	21	Exon 3	c.229C>T	p.R77W	4/44	N/A	N/A	CM991015	0.00001997
	22	Exon 3	c.229_230insGGGTGAGTAG	p.I79Sfs*5	1/44	N/A	N/A	CM991015	N/A
	23	Intron 3	c.231+1G>T	splicing	1/44	N/A	N/A	CS066305	0.00001419
	24	Exon 4	c.245G>A	p.C82Y	2/44	N/A	N/A	CM1510638	N/A
	25	Exon 12	c.936dupT	p.R313Sfs*30	1/44	N/A	N/A	CM991019	N/A
	26	Exon 12	c.937C>T	p.R313*	6/44	N/A	N/A	VCV00038870	0.00003538
	27	Exon 13	c.1079T>G	p.V360G	1/44	N/A	N/A	CM087558	N/A
	28	Exon 15	c.1288C>T	p.R430*	2/44	N/A	N/A	CM139564	0.000007102
	29	Exon 15–16	Del	N/A	1/44	N/A	N/A	CG091566	N/A
	30	Exon 22	c.2002G>A	p.G668R	6/44	N/A	N/A	CM991025	0.00001193

^a^The reference transcript is NM_000282.3

ACMG/AMP, American College of Medical Genetics and Genomics and the Association for Molecular Pathology; HGMD, human gene mutation database; N/A, not available; LP, likely to be pathogenic; P, pathogenic

A total of 28 *PCCB* variants were detected, including 18 reported and 10 novel variants (Table 3). All the novel variants were pathogenic or likely to be pathogenic based on the ACMG/AMP standards, and uploaded to ClinVar (https://www.ncbi.nlm.nih.gov/clinvar, submission ID: SUB5596791). Four patients had homozygous *PCCB* variants (c.1228C>T, c.1127G>T, c.1316A>G and c.1283C>T). The c.838dupC was the most common *PCCB* variant, with a frequency of 12.5% (8/64 alleles), followed by c.1087T>C (frequency 9.4%, 6/64 alleles), c.1228C>T (frequency 7.8%, 5/64 alleles) and c.1283C>T (frequency 9.4%, 6/64 alleles).

**Table 3:** Details of *PCCB* variants detected in the 60 Chinese patients

Novel/ reported	No	Exon	Nucleotide alteration^a^	Protein alteration	Allele frequency	ACMG/AMP grading		HGMD/Clinvar accession	Population frequency (GnomAD, v2.1.1)
						Classification	Evidence code		
Novel	1	Exon 3	c.365T>C	p.F122S	1/64	LP	PM2, PM3, PP1, PP3, PP4	N/A	N/A
	2	Exon 4	c.410A>G	p.H137R	1/64	LP	PM2, PM3, PP1, PP3, PP4	N/A	0.000007964
	3	Exon 7	c.703A>C	p.T235P	1/64	LP	PM2, PM3, PP1, PP3, PP4	N/A	N/A
	4	Exon 8	c.800C>A	p.A267D	2/64	LP	PM2, PM3, PP1, PP3, PP4	N/A	N/A
	5	Exon 8	c.839delT	p.L280Rfs*68	1/64	P	PVS1, PM2, PP1, PP3, PP4	N/A	N/A
	6	Exon 9	Del	N/A	1/64	P	PVS1, PM2, PP1, PP3, PP4	N/A	N/A
	7	Exon 10	c.967_969delGTT	p.323delV	1/64	LP	PM2, PM4, PP1, PP3, PP4	N/A	N/A
	8	Exon 12	c.1216delG	p.G407Afs*36	2/64	P	PVS1, PM2, PP1, PP3, PP4	N/A	N/A
	9	Exon 12	c.1234G>A	p.G412S	1/64	LP	PS4, PM2, PP1, PP3, PP4	N/A	N/A
	10	Exon 13	c.1339C>T	p.L447F	1/64	LP	PM2, PM3, PP1, PP3, PP4	N/A	N/A
Reported	11	Exon 1	c.30_39del10	p.G11Sfs*51	2/64	N/A	N/A	CD024190	N/A
	12	Exon 1	c.167_179delinsC	p.56_60delinsA	4/64	N/A	N/A	CX087567	N/A
	13	Exon 3	c.331C>T	p.R111*	4/64	N/A	N/A	CM119112	0.00001988
	14	Exon 5	c.457G>C	p.A153P	1/64	N/A	N/A	CM043325	N/A
	15	Exon 5	c.493C>T	p.R165W	1/64	N/A	N/A	CM930576	0.000005873
	16	Exon 7	c.733G>A	p.G245S	3/64	N/A	N/A	VCV000638037	0.00001592
	17	Exon 8	c.838dupC	p.L280Pfs*11	8/64	N/A	N/A	CI146342	N/A
	18	Exon 9	c.894_895insC	V299Rfs*3	1/64	N/A	N/A	CM1110016	N/A
	19	Exon 10	c.1087T>C	p.S363P	6/64	N/A	N/A	CM1510640	0.00003181
	20	Exon 11	c.1127G>T	p.R376L	3/64	N/A	N/A	CM1110015	N/A
	21	Exon 12	c.1228C>T	p.R410W	5/64	N/A	N/A	CM930577	0.00002833
	22	Exon 12	c.1253C>T	p.A418V	1/64	N/A	N/A	CM087564	N/A
	23	Exon 12	c.1262A>G	p.E421G	1/64	N/A	N/A	VCV000624228	N/A
	24	Exon 12	c.1283C>T	p.T428I	5/64	N/A	N/A	CM930578	0.00002390
	25	Exon 13	c.1316A>G	p.Y439C	3/64	N/A	N/A	CM022052	0.00001591
	26	Exon 13	c.1373C>T	p.A458V	1/64	N/A	N/A	VCV000638044	N/A
	27	Exon 14	c.1495C>T	p.R499*	1/64	N/A	N/A	CM930579	0.000003980
	28	Exon 15	c.1540C>T	p.R514*	1/64	N/A	N/A	CM983750	0.000007076

^a^The reference transcript is NM_000532.5

ACMG/AMP, American College of Medical Genetics and Genomics and the Association for Molecular Pathology; HGMD, human gene mutation database; N/A, not available; LP, likely to be pathogenic; P, pathogenic

### Genotype–phenotype correlations

#### Comparison in clinical features between patients with *PCCA* and *PCCB* variants

The average age of onset was 1.5 months for patients with *PCCA* variants, and 6 months for patients with *PCCB* variants. While no statistically significant difference was found in the onset age between the two groups of patients (*P* = 0.164). There was also no significant difference in disease type, gender and metabolite levels at diagnosis, and rates of complications (except for hematological abnormalities) between patients with *PCCA* and *PCCB* variants (Table 4). The death rate of cases with *PCCA* variants was similar with those with *PCCB* variants (16.7%, 4/24 cases vs. 14.7%, 5/34 cases, *P* = 1.000).

**Table 4:** Characteristics of patients with variants in *PCCA* and *PCCB* genes

Features	Patients with *PCCA* variants (n = 24)		Patients with *PCCB* variants (n = 34)		*P* value^g^
	n	%	n	%	
Onset age (months), median (range)	1.5 (0.03–24)	–	6.0 (0.07–42)	–	0.164
Age at the last follow-up (months), median (range)	33 (2–228)	–	36 (2–180)	–	0.807
Gender (male)	16	66.7	19	55.9	0.408
Disease type (early onset^a^)	12	50.0	11	32.4	0.176
Hyperammonemia	9	0.38	16	47.1	0.469
Metabolites at diagnosis, median (range)					
Blood propionylcarnitine (μmol/L)	11.4 (4.2–22.5)	–	13.7 (4.4–49.2)	–	0.534
Blood glycine (μmol/L)	358.6 (146.0–1042.0)	–	423.4 (134.6–2933.1)	–	0.530
Urine methylcitrate (mmol/mmol Cr)	23.4 (0.3–1139.1)	–	20.0 (2.4–136.5)	–	0.742
Urine 3-hydroxypropionic acid (mmol/mmol Cr)	25.7 (0.1–1041.8)	–	26.9 (1.5–841.3)	–	0.888
Neurological involvement					
Intellectual disability	15	62.5	21	61.8	0.955
Seizures	12	50.0	13	38.2	0.373
Hearing impairment^b^	2	8.3	1	2.9	0.756
Skin lesion	5	20.8	13	38.2	0.158
Hematological abnormalities^c^	6	25.0	19	55.9	0.019
Intermittent vomiting	6	25.0	12	35.3	0.404
Liver involvement^d^	5	20.8	9	26.5	0.621
Cardiac involvement^e^	3	12.5	1	2.9	0.374
Renal dysfunction^f^	1	4.2	1	2.9	1.000
Death	4	16.7	5	14.7	1.000

^a^Early onset was defined as onset age ≤ 3 months

^b^Hearing impairment referred to bilateral delay of auditory brainstem response

^c^Hematological abnormalities included anemia, pancytopenia and thrombocytopenia

^d^Liver involvement included hepatomegaly and liver dysfunction (increased liver enzymes)

^e^Cardiac involvement included long QT syndrome and heart failure

^f^Renal dysfunction referred to acute renal failure

^g^Chi-square test and Mann–Whitney U test were used for comparisons between early- and late-onset propionic acidemia

#### Types of *PCCA* variants in patients with early- and late-onset propionic acidemia

**Fig. 1:**
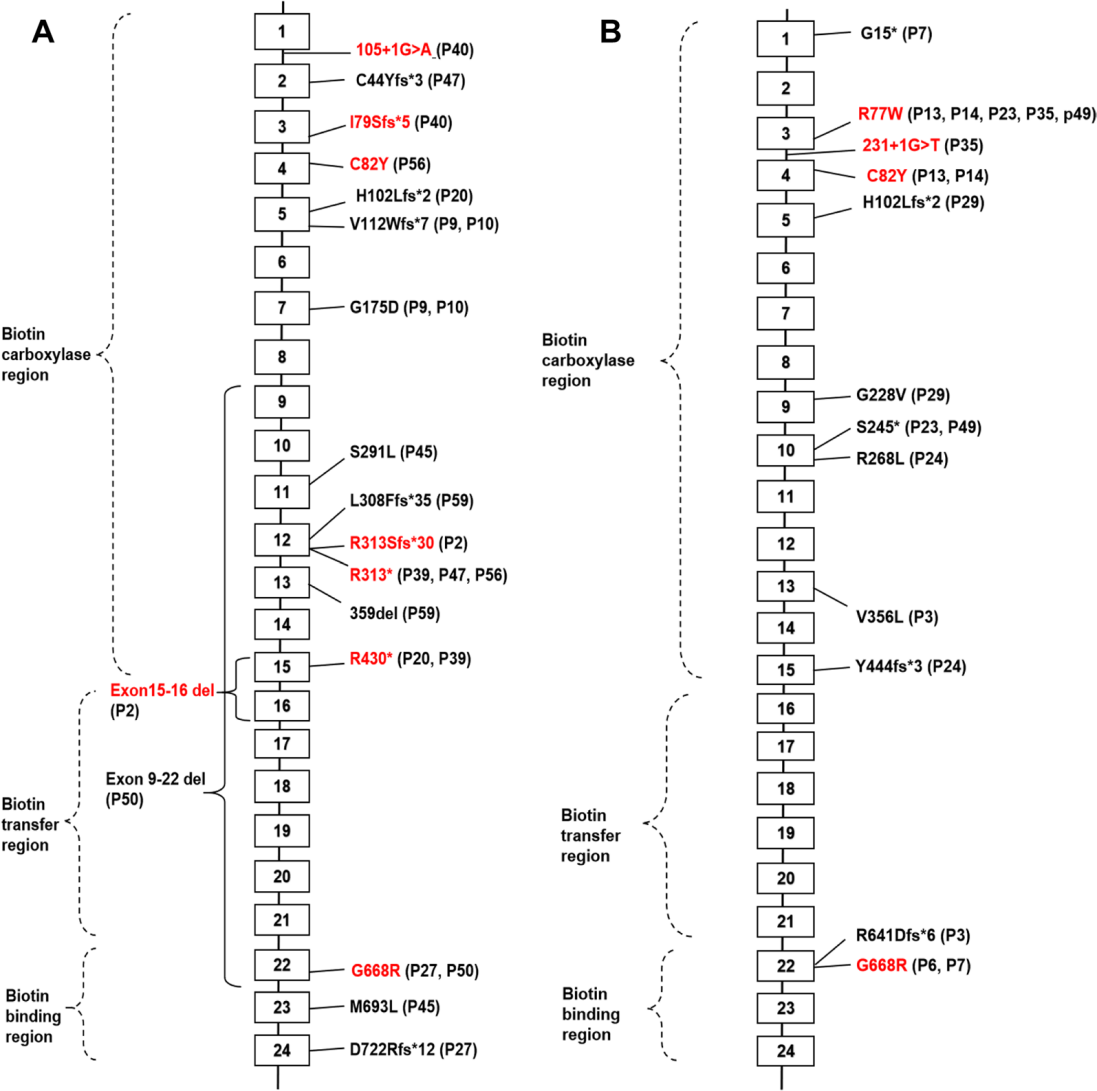
The distribution of *PCCA* variants in patients with **A** early-onset (n = 12) and **B** late-onset (n = 9) propionic acidemia. Patient’ ID was showed in the parentheses, and the reported variants were marked in red

Twelve early-onset cases had *PCCA* variants, who were all compound heterozygous. A total of 16 *PCCA* variants were detected, which were mainly distributed on exons 1–5, exons 11–13, and exons 22–24 of the 24 exons of *PCCA* gene. The frequency of c.937C>T was higher than the other 15 *PCCA* variants (Fig. 1A).

Nine late-onset cases had *PCCA* variants. One case was homozygous for c.2002G>A, and the remaining eight cases were compound heterozygous. A total of 12 *PCCA* variants were detected in the 9 cases, which were mainly distributed on exons 1–5, exons 9–10, exon 13, exon 15 and exon 22 of *PCCA* gene. Among the 12 *PCCA* variants, the most frequent variant was c.229C>T, followed by c.2002G>A (Fig. 1B).

Besides, we compared the frequency of missense, frameshift, nonsense and splicing variants between the patients with *PCCA* variants of early-onset and late-onset propionic acidemia. However, there was no significant difference in the frequency of variant types between the two groups of patients (Table 5).

**Table 5:** Types of gene variants in patients with early-onset and late-onset propionic acidemia

Gene	Type of variant	Early-onset (n = 23)^a^		Late-onset (n = 30)		*P* value^b^
		Allele number	Allele frequency (%)	Allele number	Allele frequency (%)	
*PCCA*	Missense	7	15.2	11	18.3	0.672
	Frameshift	8	17.4	3	5.0	0.054
	Nonsense	5	10.9	3	5.0	0.289
	Splicing	1	2.2	1	1.7	1.000
	Other	3	6.5	0	0	–
*PCCB*	Missense	14	30.4	22	36.7	0.502
	Frameshift	5	10.9	14	23.3	0.097
	Nonsense	1	2.2	5	8.3	0.230
	Splicing	0	0	0	0	–
	Other	2	4.3	1	1.7	0.578

^a^23 out of the 25 patients with early-onset propionic acidemia had genetic testing

^b^Chi-square test was used for statistical analysis

#### Types of *PCCB* variants in patients with early- and late-onset propionic acidemia

**Fig. 2:**
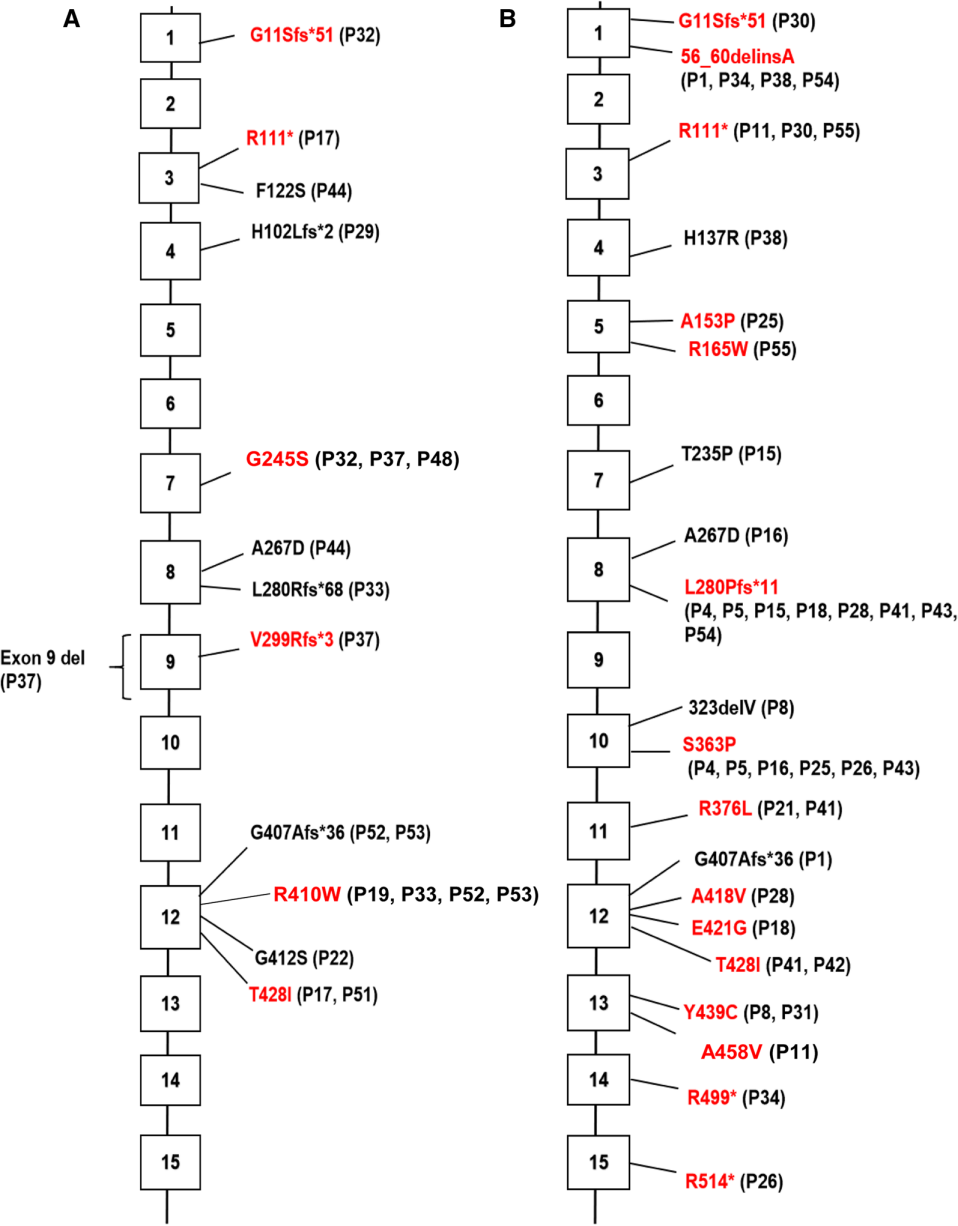
The distribution of *PCCB* variants in patients with (**A**) early-onset (n = 11) and (**B**) late-onset (n = 21) propionic acidemia. Patient’ ID was showed in the parentheses, and the reported variants were marked in red

Eleven early-onset cases had *PCCB* variants. Two cases were homozygous for *PCCB* variant c.1228C>T and c.1283C>T, respectively. A total of 12 *PCCB* variants were detected, which were distributed on exon 1, exon 3, exon 4, exons 7–9 and exon 12 of the 15 exons of *PCCB* gene. Among them, c.1228C>T on the exon 12 of *PCCB* gene was the most frequent variant (Fig. 2A).

Twenty-one late-onset cases had *PCCB* variants. Two cases were homozygous for *PCCB* variant c.1127G>T and c.1316A>G, respectively. A total of 20 *PCCB* variants were detected in the 21 patients, which were scattered on the 15 exons of *PCCB* gene. Among them, c.838dupC on exon 8 of *PCCB* gene was the most frequent variant (Fig. 2B).

We also compared the frequency of missense, frameshift, nonsense and splicing variants between the patients with *PCCB* variants of early-onset and late-onset propionic acidemia. However, no obvious difference was found in the frequency of variant types between the two groups (Table 4).

## Discussion

Propionic acidemia is a rare and serious inherited metabolic disease, with high mortality and disability. We conducted a clinical and genetic study on 60 patients from 16 provinces in mainland China, which offers relatively comprehensive and complete data on the phenotype and genotype of Chinese patients with propionic acidemia, and also provides a reference for the disease prevention and management in China.

Since newborn screening for propionic acidemia was carried out late in China, the majority of patients in our cohort were clinically diagnosed after onset, and only 5.8% (5/60) cases identified through newborn screening. A European study on 55 propionic acidemia patients showed a newborn screening rate of 36.4% [8]. Since newborn screening can find out patients at early stage of the disease and initiate timely treatment, it may reduce neurological sequelae and mortality in patients with propionic acidemia [8, 12]. Consistently, five patients detected by newborn screening in our study had relatively favorable outcome during the follow-up. With newborn screening becoming more widely available in China, more patients can benefit from early diagnosis and intervention.

Neurological involvement is a common complication of propionic acidemia. Our study showed that 60% of the patients had intellectual disability, and 41.7% had seizures, which was consistent with previous studies [19–23]. The brain imaging findings are non-specific. Basal ganglia damages were the most common abnormalities in our study. Besides, patients’ intelligence quotient may be negatively correlated with the number of metabolic decompensation episodes [8], so we should pay attention to the prevention and management of acute decompensation. In addition, 4 patients (6.7%) developed cardiac involvement in our cohort. One patient suddenly died of long QT syndrome at 19 years old. It is reported that 9–23% of propionic acidemia patients had a history of cardiac involvement (such as cardiomyopathy and long QT syndrome) [22, 24], which is one of the leading causes of death for patients with propionic acidemia besides acute metabolic decompensation [25, 26]. In addition, patients may also develop osteoporosis, skin damage, impairment of hearing, optic nerve damage, mental disorders, and other complications [8, 27, 28]. Therefore, regular and multi-disciplined assessments to prevent serious complications are necessary. It is also suggested to examine leucine to valine and/or isoleucine ratios to avoid the imbalance of branched-chain amino acids and guide the intake of medical foods [29]. Liver transplantation is recommended for the patients with frequent metabolic decompensation [30]. After liver transplantation, the clinical symptoms and protein tolerance can be significantly improved, as observed in the three liver transplanted cases in this cohort.

Previous studies reported that over 80% of propionic acidemia patients were early-onset, most of whom had clinical symptoms in the neonatal period [12, 21]. However, a low proportion (41.7%) of early-onset propionic acidemia was found in this study, which may be related to the selection bias due to the deaths of some undiagnosed early-onset patients. Previous studies have proved that early-onset patients had higher mortality, while late-onset patients had better prognosis, including less neurocognitive impairment and fewer episodes of metabolic decompensation [31]. Similarly, our findings showed a seemingly higher death rate of early-onset patients than that of late-onset patients (28% vs. 6.7%), although the difference didn’t reach the statistical significance. However, there was no significant difference in the incidences of complications between the two groups of patients, which may be explained by the short time of follow-up for patients with early-onset propionic acidemia.

*PCCA* (located on chromosome 13q32) contains 24 exons and encodes 728 amino acids. *PCCB* (located on chromosome 3q13.3-q22) contains 15 exons and encodes 539 amino acids. There is obvious difference in the gene variant spectrum of propionic acidemia patients from different countries and races [32, 33]. So far, no hotspot variants of *PCCA* or *PCCB* genes have been found in Chinese patients. In the 10 patients previously reported in Taiwan, c.1301C>T and c.-4156_183+3713del in *PCCB* were two relatively frequent variants [34], which were not consistent with the results reported in mainland China [35, 36]. In this study, no significant difference was found in the proportion of patients with *PCCA* and *PCCB* variants. c.937C>T and c.2002G>A were the most common *PCCA* variants in our cohort, both with a frequency of 13.9%. The c.2002G>A variant has been reported in Chinese [35, 36] and American [37] patients. c.838dupC (allele frequency 12.5%) and c.1087T>C (allele frequency 9.4%) were common *PCCB* variants in our cohort, which were previously reported in Chinese patients [34, 38].

Propionyl-CoA carboxylase consists of α and β subunits. The α subunit mainly contains three functional domains of N-terminal biotin carboxylase region, biotin transfer region and C-terminal biotin binding region, while the β subunit forms the core of propionyl-CoA carboxylase [4, 39]. It is considered that the relationships between the type of gene variants, the distribution of variants, the enzyme activity and the phenotype of propionic acidemia are still not very clear [4], which is accordant with our findings. The 30 *PCCA* variants detected in our study were mainly located in the N-terminal biotin carboxylase region and the C-terminal biotin binding region of α subunit. While there was no significant difference of the *PCCA* variant distribution in the functional domains between early-onset and late-onset patients. The 28 *PCCB* variants detected in our study were scattered on the exons of *PCCB* gene. There was also no significant difference of the *PCCB* variant distribution between early-onset and late-onset patients. In addition, the clinical manifestations of patients with *PCCA* variants were not dramatically different from those of patients with *PCCB* variants.

Previous studies suggest that nonsense variants are associated with severe clinical phenotypes, while some missense variants may lead to protein loss due to abnormal peptide folding, which makes the pathogenicity of missense variants difficult to predict [22, 40, 41]. Our findings showed that no difference was found in the types of gene variants between early-onset and late-onset patients, but certain variants may be associated with early-onset or late-onset propionic acidemia. In our cohort, the nonsense variant c.937C>T in *PCCA* was common in early-onset patients but absent in late-onset patients, which indicates an association with severe clinical phenotype. Missense variants c.1228C>T and c.1283C>T in *PCCB* were frequent in early-onset patients, and their corresponding homozygotes were also found in early-onset patients, which may indicate correlations with severe phenotypes. Moreover, three homozygous variants c.2002G>A in *PCCA*, c.1127G>T and c.1316A>G in *PCCB* found in the late-onset patients, two variants c.229C>T in *PCCA* and c.838dupC in *PCCB* frequent in the late-onset patients, may be related to mild clinical phenotypes. However, since most patients harbor compound heterozygous variants, it is difficult to estimate the pathogenicity of a single variant, which makes it difficult to study the correlation between clinical phenotypes and genotypes.

## Conclusions

The clinical manifestations of patients with propionic acidemia are complex and lack of specificity, and most patients have varying degrees of neurological sequelae. Newborn screening can find patients at the early stage of disease, which can be beneficial to improve clinical outcomes. In our study, a total of 19 novel *PCCA* variants and 10 novel *PCCB* variants were identified. Although the correlation between the clinical phenotype and genotype of propionic acidemia isn’t very clear, certain variants in *PCCA* or *PCCB* may be associated with early-onset or late-onset propionic acidemia. Our study further delineated the heterogeneous phenotypes and expanded the spectrum of genotypes in propionic acidemia patients.

## Supplementary Information

**Additional file 1.** An Excel sheet (.xls) named “Detailed data of every patient” describes the detailed information on biallelic variants and corresponding clinical characteristics of every patient with propionic acidemia in this study.

## Data Availability

The datasets during and/or analysed during the current study available from the corresponding author on reasonable request.
